# Potential of cold plasma pretreatment for preserving biochemical attributes and ensuring the microbiological safety of saffron stigma

**DOI:** 10.1002/fsn3.4252

**Published:** 2024-07-23

**Authors:** Zohreh Birjandi Toroghi, Razieh Niazmand, Farid Moradinezhad, Hassan Bayat

**Affiliations:** ^1^ Department of Horticultural Sciences, Faculty of Agriculture University of Birjand Birjand Iran; ^2^ Department of Food Chemistry Research Institute of Food Science and Technology (RIFST) Mashhad Iran

**Keywords:** antioxidant, cold plasma, crocin, RSM, safranal

## Abstract

Saffron, similar to numerous other agricultural commodities, is susceptible to microbial contamination during cultivation and postharvest handling. Cold plasma treatment has emerged as an effective method for microbial inactivation while preserving food quality. The aim of this research was to preserve the color integrity and minimize the presence of microorganisms in dried saffron stigma by implementing cold plasma pre‐treatment. Process parameters were optimized using the response surface method (RSM), considering the type of atmosphere (argon and air), plasma exposure time (1, 5, and 10 min), and plasma power (40, 70, and 100 W) as independent variables. The objectives were to maximize crocin content and minimize the total microbial load. The analysis of the response surface revealed that the argon atmosphere had a more significant impact on reducing microbial contamination than air, and an increase in plasma exposure time led to a decrease in microbial load. The maximum reduction in microbial load, by 0.9 logarithmic cycles compared to the control, was achieved with a 10‐min treatment at 40 W power. Extended plasma exposure durations led to a minor reduction in the color, taste, aroma, and antioxidant properties of saffron stigma. Specifically, the color, taste, and aroma decreased by 0.5%, 0.5%, and 0.08%, respectively, with longer plasma exposure times. The antioxidant activity decreased by 0.64% with prolonged exposure time. However, the plasma‐treated samples did not show any signs of *Escherichia coli*, mold, or yeast. Furthermore, our findings demonstrated that the type of atmosphere significantly influenced the reduction of infection and maintenance of saffron stigma's color quality. Cold plasma pretreatment holds promise as a viable method for preserving the physicochemical attributes of saffron while effectively reducing microbial contamination.

## Graphical abstract

Process parameters were optimized using the response surface method (RSM), considering the type of atmosphere (argon and air), plasma exposure time (1, 5, and 10 min), and plasma power (40, 70, and 100 V) as independent variables. Our findings demonstrated that the type of atmosphere significantly influenced the reduction of infection and maintenance of saffron stigma's color quality. Cold plasma pretreatment holds promise as a viable method for preserving the physicochemical attributes of saffron while effectively reducing microbial contamination.

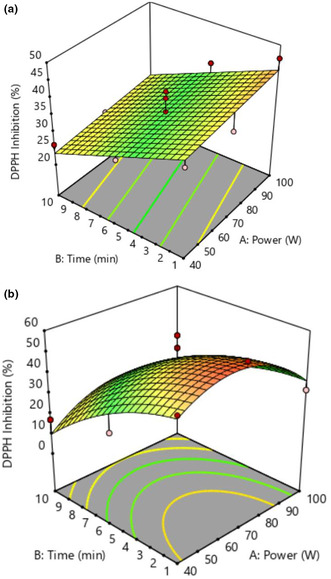

## 1 INTRODUCTION

Saffron is a perennial plant, a herb of the Iridaceae family. It is also known as red gold and is the most expensive spice worldwide. Saffron includes 1500 species and is distributed in the semi‐arid and southwestern regions of Asia (Mousavi & Bathaie, 2011). Iran is the world's leading producer of saffron, producing 430 tons in 2019. In that year, India was the second‐largest saffron producer, with only 22 tons of production (Shahbandeh, 2020). Due to the COVID‐19 pandemic and the Russia–Ukraine War Influence, the global market for organic saffron, estimated at USD 292.1 million in the year 2022, is projected to reach a revised size of USD 359 million by 2028, growing at a CAGR of 3.5% during the forecast period 2022–2028 (Food Business Insights, 2023). The most critical and effective ingredients of saffron stigma include crocin, picrocrocin, and safranal. Crocin, a rare natural carotenoid, is water‐soluble and gives saffron stigma its distinctive yellow and orange hues, constituting approximately 80% of the spice (Ahmadian et al., 2019). Picrocrocin is the second component responsible for the bitter taste of saffron stigma, which accounts for 1.13% of dry saffron stigma (Christodoulou et al., 2015). Safranal is the third principal component responsible for the aroma and smell of dry saffron stigma, and the concentration of safranal depends on the conditions of drying and storage of saffron (Shahi et al., 2016). In addition, due to its aroma, taste, and coloring, it has many applications in the food industry as a spice, as well as in the cosmetics, health, and pharmaceutical industries (Ahmadian‐Kouchaksaraie et al., 2016; Wang et al., 2011). Modern pharmacological investigations suggest that saffron has numerous therapeutic impacts on different parts of the body, namely the central nervous system as anti‐Alzheimer, anti‐Parkinson, antidepressant, and anticonvulsant, cardiovascular system, immune system, genital system, and eye. Moreover, saffron can help with cancer, cerebrovascular illnesses, diarrhea, measles, bronchitis, urinary tract infections, cough, and stomach issues (Nissar et al., 2022).

Saffron has been exposed to various microbial contaminations that enter the plant in different ways, such as soil, water, dust, and heavy metals (Moladost & Hanifian, 2016). Due to the exposure of saffron to various types of infection and the lack of suitable handling and storage conditions, the quality of the product is reduced (Hoshyar et al., 2015). Various technologies used for microbial disinfection of spices include gamma irradiation, superheated steam treatment, and fumigation with ethylene oxide, and propylene oxide. Nevertheless, these methods have drawbacks such as low consumer acceptance, negative impacts on color, flavor, and nutritional qualities, as well as the production of carcinogenic by‐products. Given these limitations and the growing demand for safe and high‐quality foods, developing new decontamination processes is essential. Cold plasma (CP) technology could serve as a promising alternative for the decontamination of herbs and spices (Darvish et al., 2022).

Cold plasma is an emerging non‐thermal technology that is used for microbial disinfection in agriculture and food industries, from collection to packaging, to preserve the nutritional value, color, and quality of foods (Darvish et al., 2022; Farooq et al., 2023) Plasma is specified as the most universally abundant state of the substance. Plasma combines reactive particles and neutral species, comprising molecules, atoms, ions, free radicals, electrons, photons, and irradiated heat created by applying energy to a neutral gas or a gas mixture (Namjoo et al., 2022). It is also capable of inactivating microorganisms such as *Escherichia coli*, *Salmonella typhimurium*, and *Staphylococcus aureus* that exist in food (Coutinho et al., 2018). The advantages of this method include reducing costs, preserving the appearance of food, reducing the use of preservatives, and improving plant performance (Darvish et al., 2022). Moreover, cold plasma does not leave toxic residues, making it an environmentally friendly postharvest treatment (Rao et al., 2023).

Research on the effect of cold plasma on food quality is limited and primarily focused on reducing food microbial contamination (Pankaj et al., 2018). Limited studies have investigated the impact of cold plasma on fresh saffron, revealing practical implications on microbial load (Darvish et al., 2022) and adverse effects on color indexes (Hosseini et al., 2018). Nevertheless, there is a lack of research on the effects of cold plasma on dried saffron. Following harvest, saffron must be promptly dried to extend its shelf life. Consequently, utilizing cold plasma treatment post‐drying may be more feasible for farmers and manufacturers due to the high production volume and time constraints.

This study aimed to determine the optimal process parameters, in terms of the type of atmosphere, exposure time, and voltage, through the response surface method to develop a model for extracting effective compounds and reducing the amount of dried saffron contamination. The optimal experimental findings were used to validate the most effective model by comparing the predicted results with the actual results. Additionally, the surface diagrams generated by the best model were utilized to investigate the mutual effects of the components.

## 2 MATERIALS AND METHODS

### 2.1 Materials

High‐quality dried saffron stigma was obtained from the farmers of the Khein Arab region (Mashhad, Razavi Khorasan, Iran). DPPH, methanol, Folin–Ciocalteu reagent, and other chemicals were supplied from stigma Co. Culture media, including Plate Count Agar (PCA) and Yeast Glucose Chloramphenicol Agar (YGC), were purchased from Merck (Darmstadt, Germany). All materials were of analytical grade.

### 2.2 Cold plasma treatments

Plasma treatment was done using a cold plasma device (Plasma, Cogarade model, made in Iran and Korea). The argon gas discharge was utilized, flowing between the two electrodes at a rate of 1 L/min. An ionizing potential was applied across a 1 cm gap between electrodes of a specific shape, resulting in the creation of a plasma arc within a Teflon cowling. The samples underwent treatment using an atmospheric pressure plasma jet with power settings of 40, 70, or 100 watts and plasma exposure durations of 1, 5, or 10 min, at a distance of 3 cm and ambient temperature. Pure argon and air served as the working gases. To assess the impact of CP on chemical and microbial properties, 2 g of dried saffron stigma were placed in a sterile glass petri dish and exposed to CP treatment. An untreated sample was used as a control to examine the effects of CP treatment on dried saffron stigma. The samples were subsequently placed in metal containers with covers in the fridge, followed by chemical and microbial analyses.

### 2.3 Determination of microbial load

One gram of weighed saffron sample was mixed in 9 mL of diluted solution. For each sample, an appropriate decimal dilution (0.1 and 0.01) was prepared to count the following microorganisms:

1. The total microbial load was carried out according to the Iranian National Standard number 5689 (2018). After making successive dilutions, pour 1 mL of the solution into the plate, and then add the culture medium of the Plate Count Agar (PCA) to the sample and invert the plates for 3 days inside an incubator at 30°C.
2. To count mold and yeast, Yeast Glucose Chloramphenicol Agar (YGC) culture medium was used, and then the plates were placed upside down in an incubator at 25°C for 5 days. After 5 days, the colonies were counted.
3. Also, *Escherichia coli* Broth (ECB) culture medium was used to count *Escherichia coli* according to the Iranian National Standard number (2946). One milliliter of the solution was cultured on the surface and kept in an incubator for 24 h at 37°C.

### 2.4 Determining the amount of crocin, picrocrocin, and safranal

The test method was according to the Iranian National Standard number 259‐2 (2013). 0.5 g of ground saffron was transferred into a 1000‐mL flask, and then 900 mL of distilled water was added to it, and stirred with a stirrer for 1 h in the darkness at a speed of 1000 rpm. After that, it was brought to a volume of 1000 mL with distilled water, and 20 mL of the solution was transferred to a 200 mL flask and made up again. The solution was filtered in the darkness, and the absorbance of crocin, safranal, and picrocrocin was read by a spectrophotometer (Dr 500 Hach model, Made in America) at wavelengths of 440, 330, and 257 nm, respectively. The percentage of crocin, picrocrocin, and safranal based on the dry matter of the sample was obtained from Equation 1.

(1) $$A=\left(D\times 10,000\right)/\left(M\times \left(100-W_{\mathrm{MV}}\right)\right)$$

where *D* = absorption of each active ingredient (crocin, safranal, and picrocrocin), *M* = sample weight, *W*_MV_ = sample moisture, and *A* = maximum absorption.

### 2.5 Determining the antioxidant activity

0.15 mM DPPH methanolic solution was prepared. 0.5 mL of the extract was mixed with 4 mL of DPPH solution. A blank sample was prepared in the same way, with the difference that 0.5 mL of methanol was mixed with 4 mL of DPPH solution instead of the extract. After 30 min in the darkness, the absorption values of the solutions were recorded by a spectrophotometer at a wavelength of 517 nm. Equation 2 was used to calculate the antioxidant activity percentage of the samples (Niazmand & Razavizadeh, 2021).

(2) $$\text{Percentage of DPPH radical inhibition}=\left(A_{b}-A_{s}\right)/A_{b}\times 100$$

where *A*_b_ = absorption of the blank and *A*_s_ = absorption of the sample.

### 2.6 Microstructure

Photos taken with a scanning electron microscope were prepared at the Department of Boali Mashhad Research Institute. To coat the equivalent of 1 g of dried saffron stigma with gold, a vacuum coating machine and argon gas were utilized. The unwrapped (control) and wrapped samples were photographed using a scanning microscope from Carl Zeiss SMT Ltd. in Cambridge, England. Multiple images were captured at magnifications ranging from 500 to 1000 times. The distance between the camera probe and the sample was adjusted between 8 and 12 mm, while an accelerating voltage of 20 kV and a secondary electron detector were employed (Taglienti et al., 2011).

## 3 STATISTICAL ANALYSIS

Statistical analysis of data and drawing of graphs were done using Design Expert version 7 software (State‐Ease Inc., Minneapolis, MN, USA). Central composite design (CCD) was used to investigate the effect of three independent variables, treatment time in three levels (1, 5, and 10 min), power in three levels (40, 70, and 100 W) and gas type in two levels (argon and air). The Design‐Expert 7 software was used to generate 26 runs, which were performed for each atmosphere with 13 treatments (8 experiments and 5 central replication points). The optimal conditions were determined with the aim of achieving the highest amount of crocin and the lowest total microbial load using the Respond Surface Methodology (RSM). Equation 3 was used to optimize the best treatment.

(3) $$Y=\beta_{0}+\sum \mathrm{ki}=1\;\beta_{i}\;\mathrm{xi}+\sum \mathrm{ki}=1\;\beta_{\mathrm{ij}}\;\mathrm{xi}2+\sum k1<i<1\;\beta_{\mathrm{ij}}\;\mathrm{xi}\times j$$

where *K* variables, *β*_0_ constant number, *β*_*i*_ coefficient of linear parameters, *xi*, *xj* variables, *β*_*ij*_ coefficient of quadratic variables.

To choose the best model for predicting second‐ and third‐order equations, they fitted from the experiments. Statistically, the best model has a probability less than .05 (*p* < .05) and the non‐significance of fit test confirms the model's inaccuracy. It should also have an explanatory coefficient (*R* ^2^) and an adjusted explanatory coefficient (*R* ^2^_Adjusted_) higher than .85 and a low predicted explanatory coefficient (*R* ^2^_Predicted_). After that, three‐dimensional graphs and counters are drawn for the desired traits. Finally, to verify the accuracy of the test and the results obtained from the model in optimal conditions, all the tests were repeated on the optimal sample and compared with the predicted results.

## 4 RESULTS AND DISCUSSION

### 4.1 Total microbial load

**FIGURE 1:**
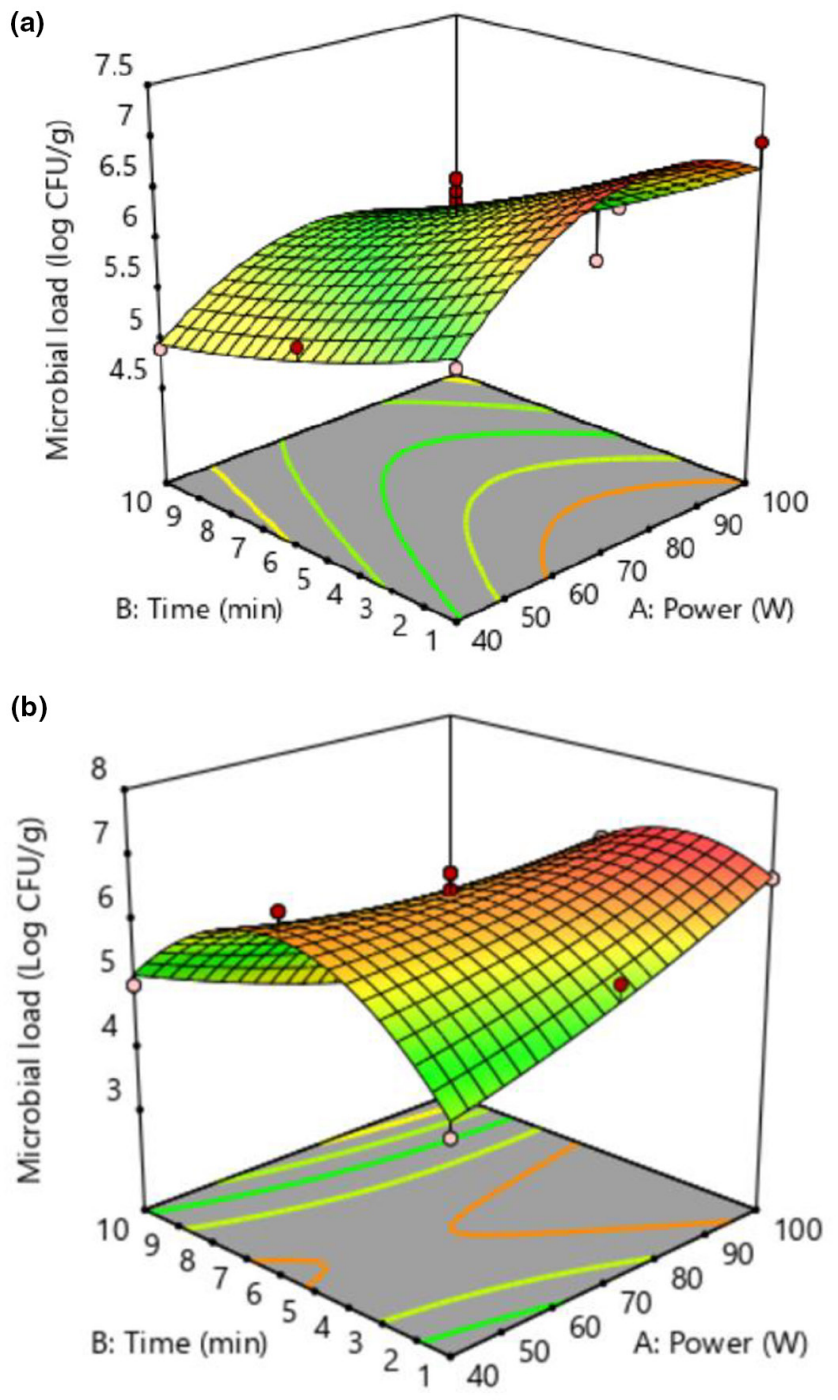
3D diagram of the impact of cold plasma on total microbial load under argon (a) and air (b) atmospheres.

The three‐dimensional diagram of the effect of power and time of plasma application on the logarithm of total microbial load in an argon atmosphere and the air is shown in Figure 1a,b, respectively. According to Figure 1a, as the time of cold plasma application increased, the total microbial count decreased. Nevertheless, despite the rise in power, a consistent pattern was not detected. In Figure 1b, the microbial load increased with increasing plasma power. However, with the rise of plasma time up to 5 min, the microbial load increased and then decreased.

The largest reduction in microbial count was noted within a 10‐min timeframe using 100 W power in an air atmosphere, resulting in a decrease of 5 logarithmic CFU/g. Plasma radiation generates electron‐excited species that can penetrate cell walls, causing harm to nucleic acids, membrane lipids, proteins, and DNA strands. This damage ultimately leads to cell destruction, morphological alterations, and cell death (Albertos et al., 2017). The results showed that, in different powers, with the increase in time in the argon atmosphere, a log 0.9 CFU/g decrease in the microbial count was observed. In contrast, in the air atmosphere, at constant power, and with the increase of time from 1 to 5 min, respectively, there was a log 7 CFU/g and log 3 CFU/g increase in count. After that, in 10 min of 4 logarithmic cycles, a decrease in the amount of microbial activity occurred. In a span of 10 min, a decrease of 4 CFU/g was observed. In addition, the results showed that the total microbial load increased by log 11 CFU/g and log 2 CFU/g in the argon atmosphere at 1 and 5 min treatments with increasing power from 40 to 100 W, and after that, the microbial load was deactivated entirely. Furthermore, in the air atmosphere at different times, with the increase in power from 40 to 100 W, the microbial count has increased by log 3 CFU/g. Our results are in line with the findings of Sanai et al. (2019). In a recent study, the effect of cold plasma with two atmospheres of N_2_ and O_2_ on saffron stigma was investigated (Darvish et al., 2022). The findings indicated that the presence of air atmosphere had a greater impact on reducing the microbial load compared to N_2_ or O_2_. So the maximum reduction was observed at 12 min and 18 V. Similarly, Khaja‐Ali et al. (2017) reported a quality reduction of saffron in which O_2_ increased in the working environment applied. The changes occurred because of the fluctuating impact of plasma on the peptidoglycan layer, resulting in the deactivation of bacteria. The variance and resilience of microbes are contingent upon both the inherent characteristics and the quality of the food to which the plasma is administered (Akbarian et al., 2018). 1. The ANOVA results for the impact of time and plasma power on microbial load in argon and air atmospheres are displayed in Tables 1 and 2, respectively. The fitting model was a quadratic polynomial (*p* < .05). Therefore, the high coefficient of explanation (*R* ^2^) in both atmospheres (.81 and .94) and the non‐significance lack of fit test confirm the accuracy of the model.

**TABLE 1:** Analysis of variance of the impact of cold plasma in argon atmosphere on the total microbial load.

Source	Sum of squares	df	Mean square	*F*‐value	*p*‐Value
Model (Quadratic)	3.94	5	0.7890	6	.0180
A‐power	0.3786	1	0.3786	2.88	.1337
B‐time	2.31	1	2.31	17.53	.0041
AB	0.0716	1	0.0716	0.5441	.4847
A^2^	1.12	1	1.12	8.53	.0223
B^2^	0.0281	1	0.0281	21.39	.6578
Residual	0.9210	7	0.1316		
Lack of fit	0.5876	3	0.1959	2.35	.2136
Pure Error	0.3334	4	0.0833		
Cor total	4.87	12			

**TABLE 2:** Analysis of variance of the impact cold plasma in O_2_ atmosphere on the total microbial load.

Source	Sum of squares	df	Mean square	*F*‐value	*p*‐Value
Model (Quadratic)	12.97	5	2.59	22.93	.0003
A‐power	0.0280	1	0.0280	0.2479	.6338
B‐time	2.86	1	2.86	25.24	.0015
AB	3.07	1	3.07	27.18	.0012
A^2^	0.0659	1	0.0659	0.5826	.4702
B^2^	6.42	1	6.42	56.78	.0001
Residual	0.7918	7	0.1131		
Lack of fit	0.3628	3	0.1209	1.13	.4379
Pure error	0.4291	4	0.1073		
Cor total	13.76	12			

### 4.2 Level of crocin

**FIGURE 2:**
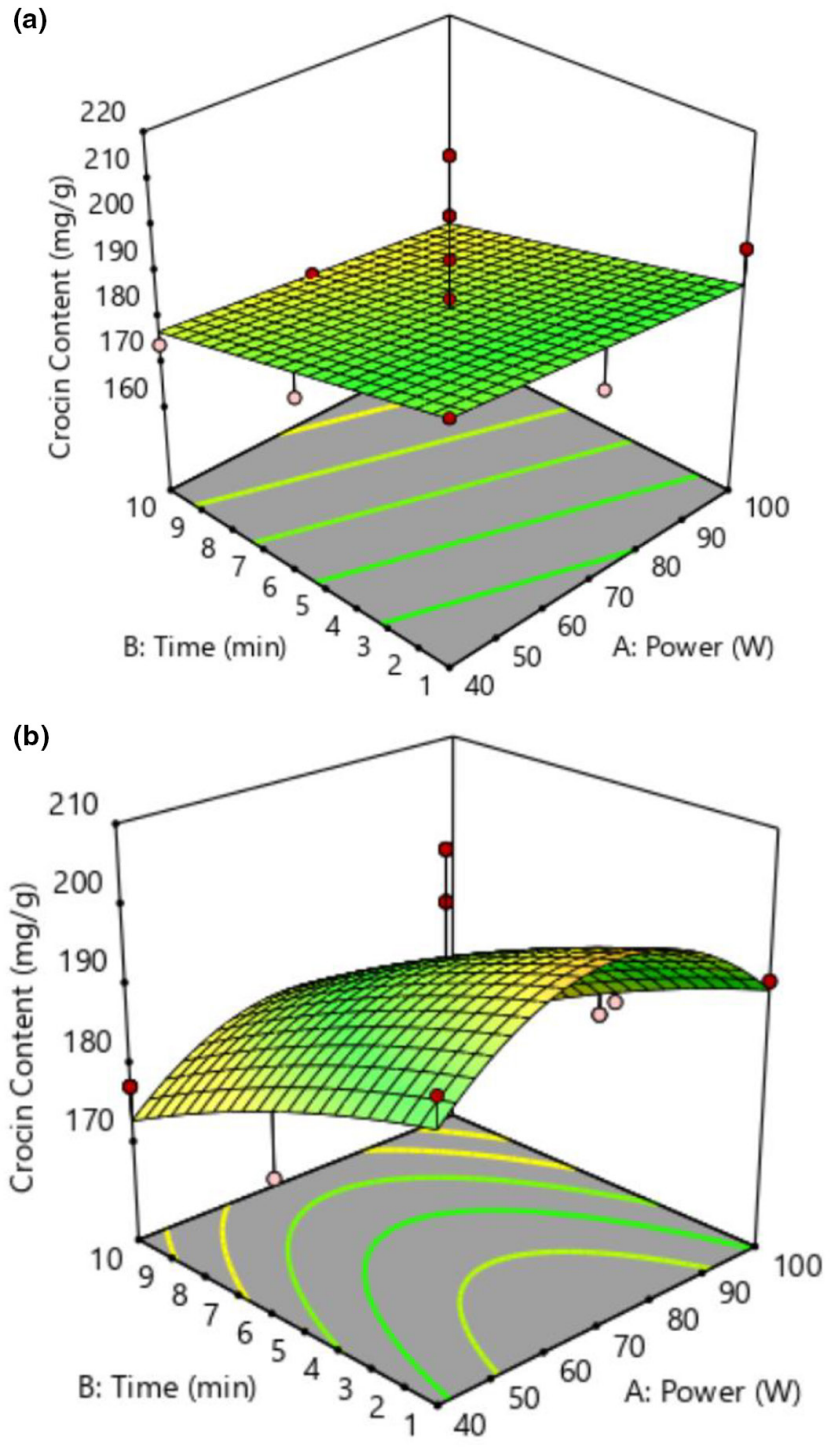
3D diagram of the impact of cold plasma on crocin content under argon (a) and air (b) atmospheres.

Figure 2 shows, the three‐dimensional diagram of the effect of power and time of plasma treatment on the crocin content in an argon (Figure 2a) and air (Figure 2b) atmosphere. According to Figure 2a, with increasing time and plasma power, no significant change in the crocin content of saffron samples was observed in both treatments (*p* > .05).

The lowest reduction rate of crocin was in 1 min and 100 W power treatment under the argon atmosphere, which decreased by 0.39%. According to Figure 2a, in the argon atmosphere and the presence of various powers, the amount of crocin decreased to 22 units with the increase in time from 1 to 10 min. In addition, at different times, with increasing power, the amount of crocin decreased by 0.8 units. The reason for the decrease in the amount of crocin during the increase in time is likely due to the sensitivity of crocin to light, and the exposure of saffron to plasma radiation can cause the destruction of cells and the loss of glycosidic bonds produced by free radicals (Akbarian et al., 2018). The results of the present research are consistent with the findings of Darvish et al. (2022).

Similarly, Hosseini et al. (2018) investigated the effect of O_2_ plasma on the secondary metabolites of saffron. They found that by raising the plasma time from 10 to 15 min, crocin decreased by 1 unit compared to the control sample. Also, in Figure 2b, the amount of crocin under air atmosphere in the presence of different powers decreased by 0.09% with increasing time from 1 to 10 min. However, in a fixed time, with increasing power, the amount of crocin did not have a constant trend, so at a fixed time of 5 min, with increasing power from 40 to 100 W, an increase of 0.028% in the crocin value was observed. After that, at the fixed time of 10 min, with the increase in power from 40 to 100 W, the crocin content decreased by 0.026%. Researchers also mentioned that enhanced input voltage reduced the quality of saffron. The saffron hue is closely tied to its crocin content, which plays a crucial role in influencing consumers due to the significant impact of product appearance. The increase of crocin during the increased plasma power can be due to the lack of contact with cold plasma particles and ultraviolet radiation, as well as the absence of unsaturated bonds in the molecular structure of crocin and its resistance to oxidizing agents (Mishra & Trivedi, 2020). The results of the effect of time and plasma power on the amount of crocin in both atmospheres are shown in Tables 3 and 4, respectively. The crocin values were not significantly affected by any of the linear, quadratic, or interaction effects of the parameters (*p* > .05). Also, the parameter of poor fit was significant for most of the mentioned models, which indicates the weak signal of the model compared to its noise. The low values of *R* ^2^, *R* ^2^_Adjusted_, and *R* ^2^_Predicted_ indicate the lack of validity and inappropriateness of the studied models for fitting crocin content data.

**TABLE 3:** Statistical results of various models on plasma power and time in argon atmosphere to predict crocin content of saffron samples.

Source	Sequential *p*‐value	Lack of fit *p*‐value	*R* ^2^	*R* ^2^_Adjusted_	*R* ^2^_Predicted_
Linear	.3717	.8990	.1796	.0155	0.1177
2FI	.9257	.8426	.1804	.0928	0.2862
Quadratic	.8473	.6804	.2183	.3401	1.5956
Cubic	.7803	.3559	.2921	.6989	17.3929

**TABLE 4:** Statistical results of various models on plasma power and time in the O_2_ atmosphere to predict the crocin content of saffron samples.

Source	Sequential *p*‐value	Lack of fit *p*‐value	*R* ^2^	*R* ^2^_Adjusted_	*R* ^2^_Predicted_
Linear	.1141	.2971	.3522	.2226	0.0417
2FI	.8929	.2392	.3535	.1381	0.2658
Quadratic	.0861	.4444	.6792	.4500	0.3050
Cubic	.8722	.1622	.6963	.2710	14.1741

### 4.3 Antioxidant activity

**FIGURE 3:**
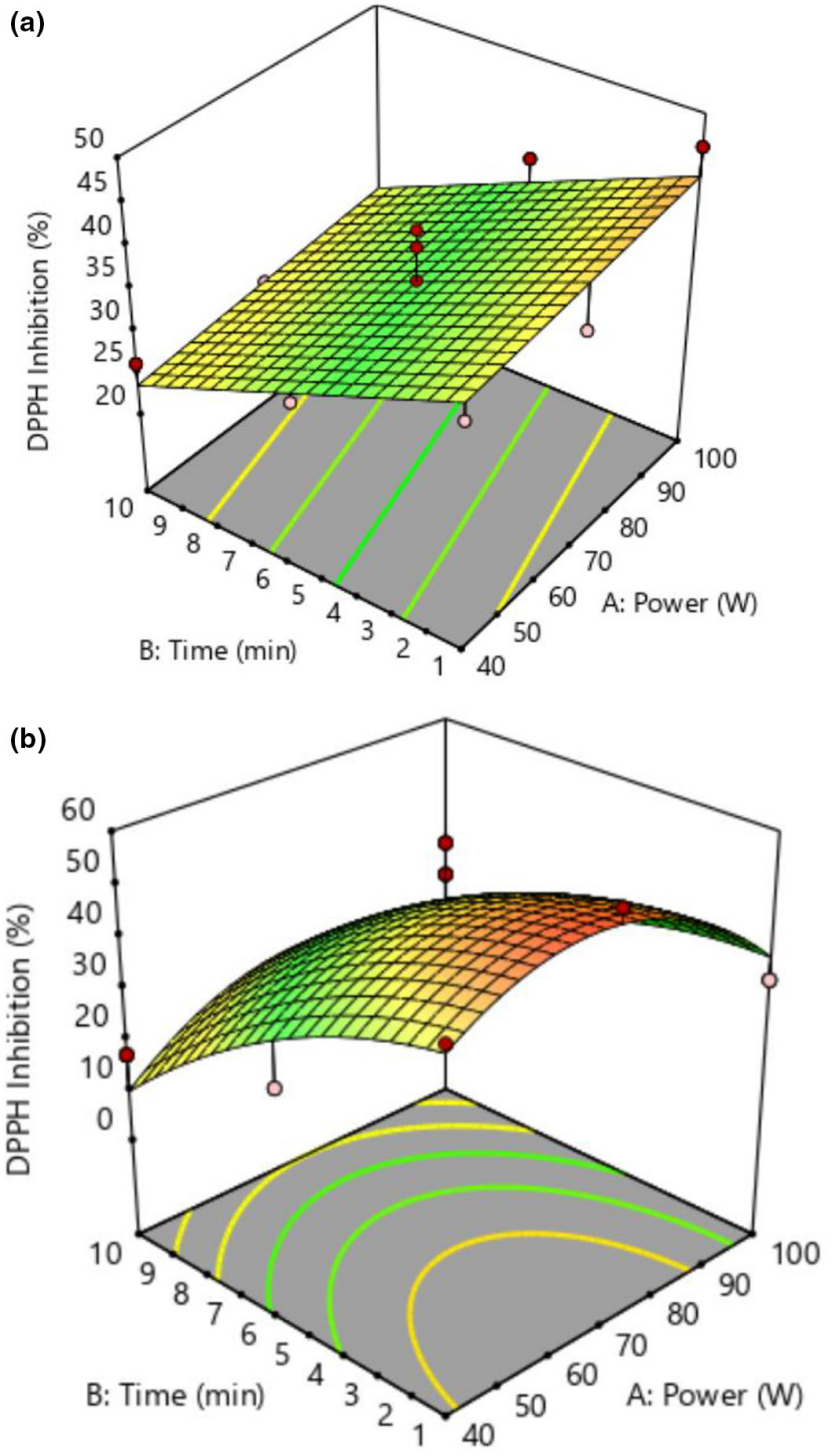
3D diagram of the impact of cold plasma on DPPH inhibition under argon (a) and air (b) atmospheres.

The three‐dimensional diagrams of the effect of power and time of plasma application on antioxidant activity under argon and air atmospheres are shown in Figure 3a,b, respectively. According to Figure 3a, with the increase in plasma power, the antioxidant activity increased. With the rise of plasma time, no significant change was observed in the antioxidant activity of saffron samples. However, in Figure 3b, with a rise in plasma power up to 70 W and plasma time up to 5 min, the antioxidant activity increased and then decreased.

The highest decrease in the antioxidant content of the air was related to the time of 10 min and the power of 100 W, which showed a decline of 0.75%. The results showed that in the argon atmosphere, increasing the power from 40 to 100 W at a fixed time of 5 min showed a 0.3 increase in the antioxidant content, while it decreased by 0.2% at a fixed time of 10 min. Also, with constant power and increasing time from 1 to 10 min, the amount of antioxidant content decreased by 0.55%. The results observed in this study were similar to the results reported by Tappi et al. (2018). It has been reported that cold plasma causes more accumulation of polyphenolic compounds such as coumaric acid and gallic acid, which increases antioxidant activity (Li et al., 2019).

The results reported by Sanai et al. (2019) on the effect of cold plasma under three types of atmospheres (O_2_, N_2_, and Ar) on the antioxidant activity of turmeric showed that with increasing plasma time from 15 to 25 min, the antioxidant activity of turmeric decreased, while the type of atmosphere did not affect the reduction rate. Moreover, in the atmosphere of air at constant powers of 40 to 100 W, with increasing time from 1 to 10 min, the antioxidant activity decreased by 18 units. While at constant times of 1 and 5 min, with increasing power from 40 to 100 W, an increase of 8 units was observed in the antioxidant activity. However, at a constant time of 10 min, with increasing power from 40 to 100 W, it decreased by 3 units. This reduction is likely due to the oxidation caused by active plasma particles and radicals, and the response of living tissue to exposure to plasma may be more complex and dependent on the time of plasma treatment (Tappi et al., 2018).

It has been reported that in 10 min, a 4% rise in the antioxidant activity of apples was observed, but with the increase of time up to 120 min, a decline of about 14.40 mmol/g was reported (Hosseini et al., 2018). The analysis of variance of the effect of time and plasma power on antioxidant activity in both argon and air atmospheres is given in Tables 5 and 6, respectively. The fitted quadratic polynomial model was significant (*p* < .05). The high coefficient of explanation (*R* ^2^) for both atmospheres is .83, and the non‐significance lack of fit test confirms the model's accuracy.

**TABLE 5:** Analysis of variance of the impact of cold plasma in argon atmosphere on DPPH radical inhibitory rate.

Source	Sum of squares	df	Mean square	*F*‐value	*p*‐Value
Model (Quadratic)	463.42	5	92.68	6.89	.0125
A‐power	21.21	1	21.21	1.58	.2497
B‐time	364.73	1	364.73	27.10	.0012
AB	54.54	1	54.54	4.05	.0840
A^2^	1.79	1	1.79	0.13	.7263
B^2^	22.68	1	22.68	1.68	.2354
Residual	94.22	7	13.46		
Lack of fit	37.92	3	12.64	0.8980	.5158
Pure error	56.30	4	14.07		
Cor total	557.64	12			

**TABLE 6:** Analysis of variance of the impact of cold plasma in the O_2_ atmosphere on the DPPH radical inhibition rate.

Source	Sum of squares	df	Mean square	*F*‐value	*p*‐Value
Model (Quadratic)	2411.41	5	482.28	7.29	.0106
A‐power	23.17	1	23.17	0.3503	.5725
B‐time	1386.85	1	1386.85	20.97	.0025
AB	43.23	1	43.23	0.6537	.4454
A^2^	531.82	1	531.82	8.04	.0252
B^2^	106.21	1	106.21	1.61	.2456
Residual	462.91	7	66.13		
Lack of fit	256.66	3	85.55	1.66	.3112
Pure Error	206.25	4	51.56		
Cor total	2874.32	12			

## 5 OPTIMIZATION OF COLD PLASMA FOR SAFFRON STIGMA

The Design Expert software and response surface method were utilized to determine the ideal cold plasma treatment conditions, taking into account the time and plasma power parameters. Considering the importance of reducing microbial contamination and preventing the reduction of saffron's color quality (crocin content), the best model was used, which was the second‐order model. The optimization was done based on the minimum microbial load and the maximum amount of crocin in the Ar and air atmospheres. The optimization was performed within the designated range of plasma power and time, taking into account their respective importance and weight, which were assigned values of 3 and 1, respectively. In addition, optimization was done with the aim of achieving the highest crocin and the lowest microbial load, with the importance of 5 and 4 and the weight of 10 and 9, respectively. The values predicted by the model are given in Table 7. To verify the model, a practical test was carried out under the predicted optimal conditions, and the characteristics of the saffron sample were measured (Table 7). As Table 7 shows, the laboratory results were similar to the predicted results by the software in both treatments, and no significant difference was observed between the atmospheres used under plasma conditions (argon atmosphere and air). Therefore, both atmospheres can be used optimally. According to the laboratory results in optimal conditions, the amount of crocin in argon treatment is higher, and its microbial load is lower. Therefore, argon treatment can be suitable. The optimized argon sample was subjected to further tests that are described below. Nevertheless, due to the increased crocin content and reduced microbial load observed in samples treated in an argon atmosphere, argon gas was selected as the optimal atmosphere for cold plasma treatment.

**TABLE 7:** Optimum conditions of variables, predicted, and laboratory responses based on crocin and total microbial load.

Atmosphere	Conditions	Crocin (mg/g)	Microbial load (CFU/g)
Argon (time 1 min, power 40 W)	Predicted	193.290^a^	1 × 10^6a^
	Laboratory	194.18 ± 0.8^a^	7 × 10^5a^
Air (time 1 min, power 70 W)	Predicted	200.883^a^	3 × 10^5a^
	Laboratory	187.47 ± 8^a^	5 × 10^6a^

*Note*: Similar letters indicate a non‐significant different for each evaluated trait according to LSD 5% probability.

### 5.1 The amount of safranal and picrocrocin for the optimal argon sample

The optimum and control values of safranal and picrocrocin are given in Table 8. In the plasma sample seen under optimal conditions, the safranal (41.87 mg/g) and picrocrocin (48.70 mg/g) values decreased by about 0.086 and 50.0% compared to safranal (45.83 mg/g) and picrocrocin (63.14 mg/g) in the control sample. Researchers investigated the effect of cold Ar plasma on the secondary metabolites of saffron. They showed that with increasing plasma power and the presence of O_2_ in Ar plasma, the amount of picrocrocin in saffron decreased (Pankaj et al., 2018). The presence of O_2_ and light with the increase in plasma time and also the reaction of active species cause the breakdown and destruction of the produced glycosidic bonds. It leads to a decrease in the amount of picrocrocin (Bourke et al., 2017). It was reported that after subjecting the crocin esters and safranal to Ar treatment at 8 kV, notable reductions were observed. The introduction of 5 and 10% O_2_ or the elevation of the input voltage resulted in further decreases in safranal and crocin esters. The decline in saffranal following the cold plasma treatment could be attributed to the oxidative degradation of saffranal, resulting in the formation of isophorene‐related compounds (Amini et al., 2017).

**TABLE 8:** Chemical and microbial characteristics of the optimal sample treated under an argon atmosphere.

Property	Optimal sample	Control sample
Safranal (mg/g)	41.87	45.83
Picrocrocin (mg/g)	48.70	63.14
Mold and yeast (CFU/g)	0	10^2^
*Escherichia coli* (CFU/g)	—	—

### 5.2 The amount of mold and yeast in the optimal argon sample

The optimal and control amounts of mildew and yeast are presented in Table 8. According to the results, the mold and yeast were significantly deactivated, as their values are lower than the National Standard of Iran (10^3^CFU/g), indicating that plasma affected mold and yeast. Darvish et al. (2022) investigated the effect of Ar plasma on the amount of mold and yeast in saffron at 5, 15, and 25 min. They reported that the count of mold and yeast significantly decreased as plasma exposure time increased. The presence of free radicals, active species, and ultraviolet photons is responsible for the decrease. These elements interact with microorganisms, resulting in the perforation of the cell wall and the accumulation and penetration of pollutants into the cell. Consequently, this leads to alterations in the morphology of the cell wall. (Darvish et al., 2022).

### 5.3 The number of *Escherichia coli* in the optimal argon sample

The *Escherichia coli* count in the saffron sample before and after plasma treatment was negative (Table 8). This result follows the National Standard of Iran, which shows the positive effect of plasma treatment on the reduction of *Escherichia coli*. Researchers investigated the impact of cold argon plasma on pumpkin puree for disinfection. The results showed that with the increase in plasma time, a significant decrease in the number of Gram‐negative *Escherichia coli* bacteria was seen, so that in 10 min, a 3.6 logarithmic cycle decrease in the number of *Escherichia coli* was observed. The findings indicated that as the duration of plasma exposure increased, there was a notable decline in the quantity of Gram‐negative *Escherichia coli* bacteria. Specifically, within a span of 10 min, there was a reduction of 3.6 logarithmic cycles in the number of *Escherichia coli*. Conversely, a decrease in the number of *Bacillus cereus* and *flavus* bacteria was observed after 20 min of exposure (Leal et al., 2018). Khodabandeh et al. (2024) utilized the response surface methodology to optimize the radiofrequency low‐pressure cold plasma processing of saffron. Their results indicated that the optimal conditions were identified as an RF power of 76 W for 26 min, resulting in reductions of total microorganisms, coliforms, *Escherichia coli*, molds, and yeast by 5.75, 6.71, 6.07, and 4.00 log CFU/g, respectively.

Reports indicate that *E. coli* bacteria are more sensitive to plasma radiation than other bacteria and are destroyed in a shorter period. The reason for the destruction of Gram‐negative bacteria is the presence of a thin cell wall (<10 nm). In comparison, Gram‐positive bacteria have a cell wall of 80 nm and are thicker (Bourke et al., 2017). Plasma disrupts the structure of peptidoglycans in the cell wall of Gram‐positive bacteria; in contrast, the inactivation of Gram‐negative bacteria can be caused by the destruction of membrane lipid layers by plasma radiation. Moreover, the practical factors in the destruction of microbes and bacteria under plasma are the presence of excited and reactive species, ultraviolet radiation, type of environment, number of cell layers, environment performance, and gases (Nishime et al., 2017).

### 5.4 Scanning electron microscope (SEM)

**FIGURE 4:**
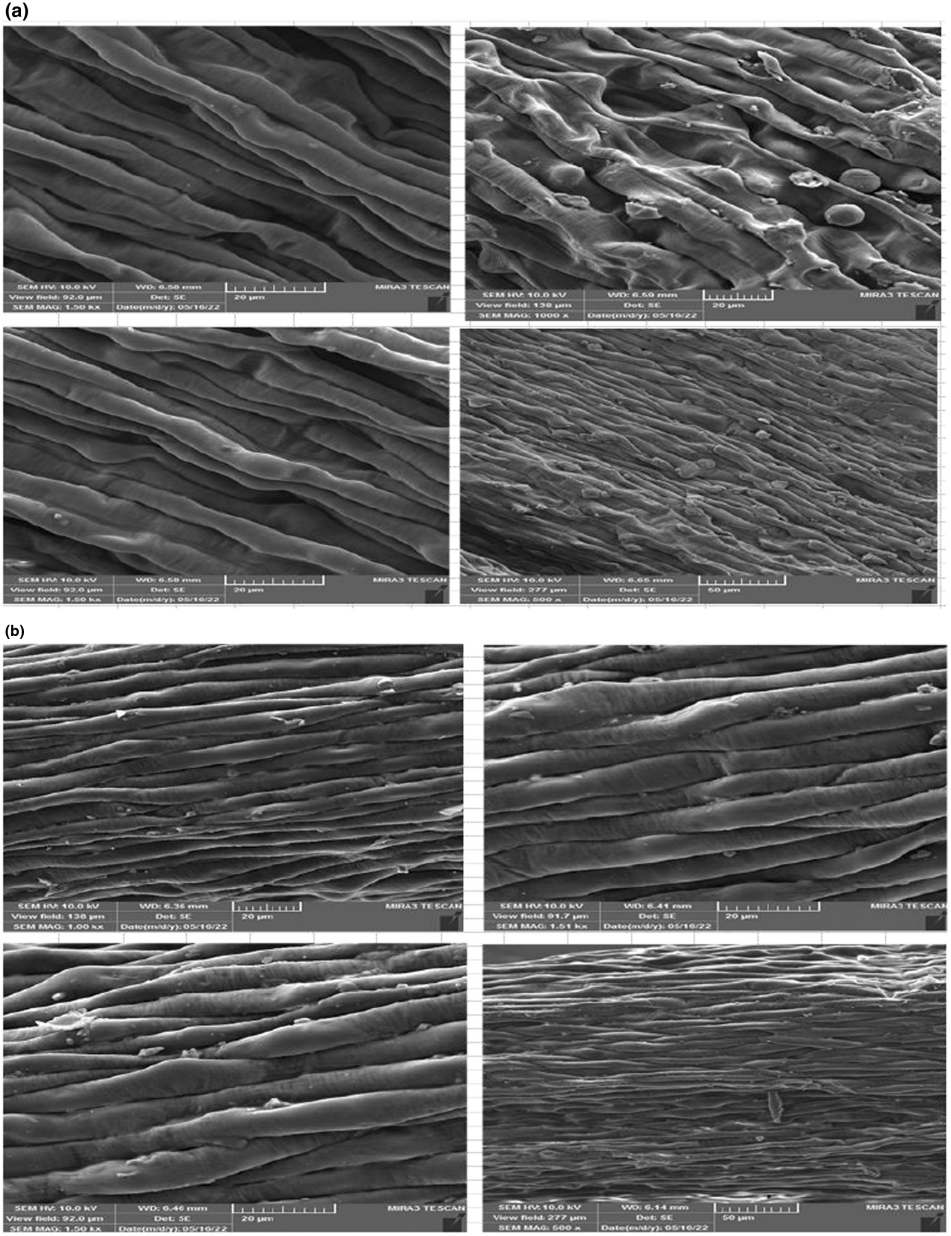
SEM images of saffron stigma strands (a) before cold plasma pretreatment (control sample) and (b) after cold plasma pretreatment (optimal conditions atmosphere argon) at various magnifications.

The SEM images of saffron stigma before and after plasma application are shown in Figure 4a,b, respectively. According to the images, the type of atmosphere used in the plasma affected the structure of the saffron stigma. According to reports, the saffron stigma particles subjected to cold plasma treatment experience alterations in their morphology. These changes involve an augmentation in chloroplast volume, disruption, and deterioration of the grana thylakoid stroma, ultimately resulting in decomposition following swelling (Terai et al., 2000). Similarly, after the application of plasma, the surface of the stigma was free of any bubbles and had a wrinkled structure. In contrast, in the sample before the application of plasma, small and large bubbles were observed on the surface of the stigma (Sinha & Panigrahi, 2010). As seen in Figure 4, the surface of the stigma in the control sample was rough, whereas in the plasma sample, the surface of the stigma became thinner. Additionally, with prolonged exposure to plasma, there was an increase in stigma degradation, leading to the development of perforations on the outer layer. Furthermore, the use of plasma pretreatment accelerated the aging process, caused discoloration of the stigma, and reduced its antioxidant properties and crocin content.

## 6 CONCLUSION

The findings revealed that treating cold plasma under argon gas resulted in the lowest microbial count and the highest concentration of crocin, making it the most effective treatment. While the safranal and picrocrocin levels slightly decreased in the argon treatment group compared to the control group, the results suggest that plasma did not have a negative impact on these compounds. Furthermore, Escherichia coli was not detected, and the levels of mold and yeast were within acceptable limits (103 CFU/g). Scanning electron microscope images showed that the stigma surface treated with plasma exhibited a wrinkled structure without any bubbles. Considering the impact of time and argon atmosphere in comparison to other factors, it is recommended to use longer treatment durations with medium power levels (within the range of this study) of cold plasma to effectively reduce microbial contamination and preserve the color of saffron stigma.

## AUTHOR CONTRIBUTIONS

**Zohreh Birjandi Toroghi:** Methodology, Investigation, Formal analysis, Writing. **Farid Moradinezhad:** Methodology, Reviewing and Editing. **Razieh Niazmand:** Methodology, Writing, Reviewing and Editing. **Hassan Bayat:** Reviewing and Editing.

## FUNDING INFORMATION

The authors would like to thank the University of Birjand for providing financial support for this project.

## CONFLICT OF INTEREST STATEMENT

The authors declare that they have no conflicts of interest.

## ETHICS STATEMENT

The authors followed the Ethical Responsibilities of Authors and COPE rules. This study does not involve any human or animal testing.

## CONSENT TO PARTICIPATE

On behalf of all co‐authors, I believe the participants gave informed consent to participate in this study.

## CONSENT FOR PUBLICATION

I, Farid Moradinezhad, give my consent for submitted manuscript to be published in *Food Science and Nutrition*.

## Data Availability

All data presented in the manuscript.
